# Longitudinal Progression of Horseshoe Retinal Tear–Associated Rhegmatogenous Retinal Detachment Without Clinically Evident Complete Posterior Vitreous Detachment: A Case Report

**DOI:** 10.1155/crop/4671416

**Published:** 2026-09-03

**Authors:** Donghyun Lee

**Affiliations:** ^1^ Department of Ophthalmology, Incheon Sejong Hospital, Incheon, Republic of Korea; ^2^ Department of Ophthalmology, Myongji Hospital, Goyang, Republic of Korea, kdmc.or.kr; ^3^ Department of Ophthalmology, Yonsei University College of Medicine, Seoul, Republic of Korea, yonsei.ac.kr

**Keywords:** horseshoe retinal tear, optical coherence tomography, posterior vitreous detachment, rhegmatogenous retinal detachment, ultrawidefield imaging, vitreoretinal traction

## Abstract

Posterior vitreous detachment (PVD) is considered a key event in the pathogenesis of rhegmatogenous retinal detachment (RRD), and horseshoe retinal tears are generally associated with vitreoretinal traction during PVD evolution. We report the longitudinal progression of horseshoe retinal tear–associated RRD without clinically evident complete PVD.

A 64‐year‐old nonmyopic man presented to a general ophthalmology clinic with floaters and preserved visual acuity. Retrospective review of baseline ultrawidefield (UWF) imaging demonstrated a horseshoe retinal tear with localized macula‐on temporal RRD extending to within approximately 2.5 disc diameters of the fovea, which had not been recognized initially. Posterior‐pole optical coherence tomography (OCT) showed posterior hyaloid attachment at the macula and optic nerve head, without clinically evident complete PVD. However, posterior‐pole OCT could not exclude localized peripheral vitreous separation or focal vitreoretinal adhesion adjacent to the tear. Approximately 6 months later, the patient presented with acute visual deterioration, and the detachment had progressed to a bullous macula‐off RRD arising from the same tear. PVD was induced during pars plana vitrectomy.

This case documents longitudinal progression of localized horseshoe retinal tear–associated RRD without clinically evident complete PVD. Although the underlying mechanism remains inferential, the clinical course is compatible with focal peripheral traction occurring before complete PVD becomes clinically evident. In symptomatic patients, preserved central visual acuity, unremarkable posterior‐pole findings, and absence of clinically evident complete PVD should not lower concern for peripheral vitreoretinal traction, retinal breaks, or RRD. Meticulous peripheral retinal examination and intentional review of the temporal retina on UWF imaging remain essential.

## 1. Introduction

Rhegmatogenous retinal detachment (RRD) is a vision‐threatening condition caused by the passage of liquefied vitreous through a retinal break, leading to separation of the neurosensory retina from the retinal pigment epithelium. Recent reviews have emphasized that timely recognition of retinal tears before progression to retinal detachment remains essential for vision preservation [1].

Posterior vitreous detachment (PVD) plays a central role in the pathogenesis of most cases of RRD by generating vitreoretinal traction and retinal tears. Recent widefield optical coherence tomography (OCT) studies have further demonstrated that horseshoe retinal tears are consistently associated with persistent anterior vitreous traction, reinforcing the pivotal role of the vitreoretinal interface in retinal tear formation [2]. However, these studies primarily describe the cross‐sectional morphology of peripheral vitreoretinal traction, whereas the longitudinal clinical evolution of traction‐related retinal tears before complete PVD becomes clinically evident, particularly in older, nonmyopic eyes, remains incompletely characterized.

RRD without clinically evident complete PVD has been described more commonly in younger, frequently myopic patients, in whom atrophic holes or retinal dialysis, rather than tractional flap tears, are the predominant causative lesions [3, 4]. This familiar phenotype differs from the present case, which involved a horseshoe retinal tear in an older, nonmyopic eye and documented progression from localized macula‐on to bullous macula‐off RRD over time without clinically evident complete PVD.

We report the longitudinal clinical course and multimodal imaging findings of this case. The report also highlights the diagnostic risk of relying on preserved central vision and posterior‐pole findings when evaluating symptomatic patients and discusses a possible, but unconfirmed, mechanism by which focal peripheral traction may occur during an incomplete or evolving stage of PVD.

## 2. Case Presentation

### 2.1. Initial Presentation at the General Ophthalmology Clinic

A 64‐year‐old man with hypertension, diabetes mellitus, dyslipidemia, and unstable angina initially presented to the general ophthalmology clinic of a secondary referral hospital on September 4, 2023. At that time, a dedicated retina clinic had not yet been established at the institution. He reported black floaters in the right eye for 2 weeks and white threadlike lines for 1 week. He had no previous ocular history.

Autorefraction demonstrated mild hyperopic astigmatism (+1.25–1.00 × 86 in the right eye and +0.75−0.50 × 95 in the left eye), without myopia. Best‐corrected visual acuity (BCVA) was 20/20 bilaterally, and intraocular pressures were within normal limits. Slit‐lamp examination showed mild bilateral cataracts.

**Figure 1:**
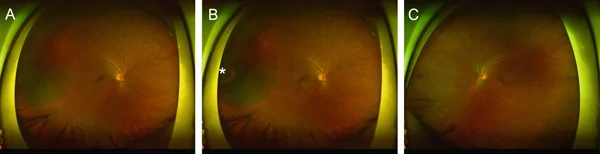
Ultrawidefield fundus images obtained at initial presentation on September 4, 2023. (A) Right eye showing a localized temporal rhegmatogenous retinal detachment (RRD) extending from approximately 8 to 10 o′clock, with a horseshoe retinal tear at approximately the 9‐o′clock position. The subretinal fluid extends posteriorly to within approximately 2.5 disc diameters of the fovea, whereas the macula remains attached. (B) Annotated image of the right eye. The shaded region outlines the extent of the localized macula‐on RRD, and the asterisk indicates the horseshoe retinal tear. (C) Left eye demonstrating no remarkable retinal abnormalities.

Nonmydriatic ultrawidefield (UWF) fundus photography was obtained at the initial general ophthalmology visit using an Optos imaging system (Optos plc, Dunfermline, Scotland, United Kingdom) (Figure 1A–C). The peripheral retinal detachment was not appreciated during the initial evaluation, and routine follow‐up was scheduled.

**Figure 2:**
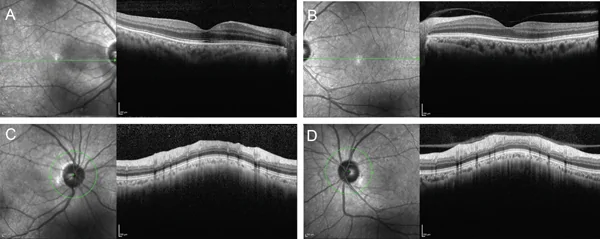
Posterior‐pole optical coherence tomography findings obtained at the initial presentation on September 4, 2023. (A, C) Right eye showing posterior hyaloid attachment at the optic nerve head and macula, with no clinically evident complete posterior vitreous detachment (PVD) within the imaged posterior‐pole region. (B, D) Left eye demonstrating partial PVD with perifoveal vitreous separation.

Posterior‐pole OCT was performed using the Spectralis system (Heidelberg Engineering, Heidelberg, Germany). The scans included the macula and optic nerve head region and showed persistent posterior hyaloid attachment at both locations in the right eye, with no clinically evident complete PVD within the imaged posterior‐pole region (Figure 2A,C; Video S1). The left eye showed partial PVD with perifoveal vitreous separation (Figure 2B,D).

### 2.2. Presentation at the Retina Clinic

**Figure 3:**
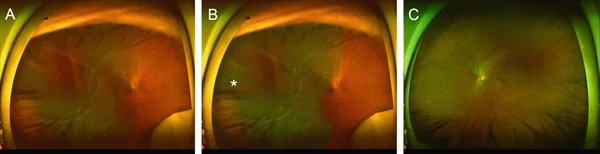
Ultrawidefield fundus images obtained at the retina clinic presentation on February 26, 2024, approximately 6 months after the initial presentation. (A) Right eye showing a bullous macula‐off rhegmatogenous retinal detachment (RRD) extending from 6 to 12 o′clock. A horseshoe retinal tear is present at approximately the 9‐o′clock position, corresponding to the lesion identified retrospectively on the baseline image. A subretinal fibrotic band suggests chronicity. (B) Annotated image of the right eye. The shaded region outlines the extent of the bullous macula‐off RRD, and the asterisk indicates the horseshoe retinal tear. (C) Left eye showing no remarkable retinal abnormalities.

**Figure 4:**
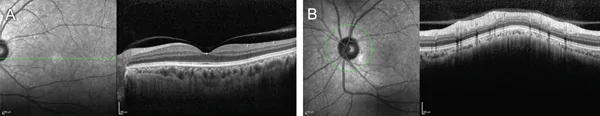
Follow‐up posterior‐pole optical coherence tomography (OCT) findings of the left eye on February 26, 2024. (A,B) Left eye showing persistent partial posterior vitreous detachment with perifoveal vitreous separation and minimal interval change compared with the initial imaging obtained approximately 6 months earlier. Posterior‐pole OCT of the right eye could not be reliably obtained because of the bullous macula‐off rhegmatogenous retinal detachment.

Approximately 6 months later, on February 26, 2024, the patient was evaluated in the newly established retina clinic at the same institution because of a sudden decrease in vision in the right eye that had begun 1 day earlier. BCVA in the right eye was counting fingers at 30 cm. Dilated fundus examination and UWF imaging revealed a bullous RRD extending from 6 to 12 o′clock and involving the macula, with a subretinal fibrotic band suggestive of chronicity (Figure 3A,B). A horseshoe retinal tear was identified at approximately the 9‐o′clock position. Mild vitreous opacities were also noted. OCT of the right eye could not be reliably obtained because of the bullous macula‐off RRD. The left eye remained unremarkable on fundus examination (Figure 3C). Follow‐up OCT of the left eye continued to demonstrate partial PVD, with minimal interval change from baseline (Figure 4A,B).

### 2.3. Retrospective Imaging Assessment and Surgical Referral

Following the diagnosis of bullous macula‐off RRD in the retina clinic, the treating retinal specialist retrospectively compared the baseline and follow‐up UWF images (Figure 1A,B). This review confirmed that a temporal horseshoe retinal tear at the 9‐o′clock position and an associated localized macula‐on RRD had already been present on the baseline UWF image. At baseline, the subretinal fluid extended posteriorly to within approximately 2.5 disc diameters of the fovea. Comparison with the follow‐up UWF image demonstrated progression from localized macula‐on RRD to bullous macula‐off RRD arising from the same horseshoe retinal tear over approximately 6 months.

Imaging assessment was modality‐specific: UWF imaging was used to establish the location of the horseshoe retinal tear and compare the extent of the detachment between the two time points, whereas posterior‐pole OCT was used to evaluate posterior hyaloid attachment at the macula and optic nerve head within the scanned region. The OCT scans did not include the peripheral vitreoretinal interface adjacent to the retinal tear and, therefore, could not exclude localized peripheral vitreous separation or persistent focal vitreoretinal adhesion.

Because pars plana vitrectomy was unavailable at our institution, the patient was urgently referred to a tertiary referral center, where pars plana vitrectomy was performed on February 27, 2024. According to the available operative record, PVD was induced intraoperatively. Detailed intraoperative findings and postoperative anatomical and visual outcomes were unavailable. The clinical course is summarized in Table 1.

**Table 1:** Clinical timeline.

Date	Clinical presentation	Key clinical and imaging findings	Vitreous status	Clinical course and management
September 4, 2023	Black floaters for 2 weeks and white threadlike lines for 1 week; BCVA 20/20; mild hyperopic astigmatism without myopia	Retrospective review performed after the February 2024 retina clinic evaluation: Baseline UWF imaging demonstrated a temporal horseshoe retinal tear at 9 o′clock with localized macula‐on RRD.	Left eye: Partial PVD	Routine follow‐up was scheduled after the initial general ophthalmology evaluation.
		Initial assessment: Localized macula‐on RRD was not appreciated.	Right eye: Posterior hyaloid attached at the macula and optic nerve head on posterior‐pole OCT; no clinically evident complete PVD within the imaged area.	

February 26, 2024	Sudden decrease in vision for 1 day; BCVA counting fingers at 30 cm	Bullous macula‐off RRD extending from 6 to 12 o′clock, with a subretinal fibrotic band; horseshoe retinal tear at 9 o′clock corresponding to the lesion retrospectively identified on baseline UWF imaging	Left eye: PartialPVD with minimalinterval change	Evaluated in the newly established retina clinic at the same institution; Urgent referral to a tertiary referral center for surgical management.
			Right eye: No clinically evident complete PVD on clinical examination; OCT could not be reliably obtained because of the bullous macula‐off RRD.	

February 27, 2024	—	PVD induced intraoperatively, according to the available operative record	Right eye: PVD induced intraoperatively	Pars plana vitrectomy performed at the tertiary referral center; postoperative anatomical and visual outcomes unavailable

Abbreviations: BCVA, best‐corrected visual acuity; RRD, rhegmatogenous retinal detachment; UWF, ultrawidefield; PVD, posterior vitreous detachment.

## 3. Discussion

Recent widefield OCT studies have substantially advanced the understanding of the morphology of peripheral vitreoretinal traction. Govetto et al. [2] demonstrated persistent anterior vitreous traction in horseshoe retinal tears using widefield OCT, reinforcing the concept that tractional flap tears are closely associated with the vitreoretinal interface. UWF swept‐source OCT studies have shown that peripheral retinal breaks may be further characterized by associated subretinal fluid, edge configuration, and, when the peripheral vitreous is adequately visualized, vitreoretinal traction [5, 6]. However, these observations are largely cross‐sectional, whereas the longitudinal clinical evolution of horseshoe retinal tears in eyes without clinically evident complete PVD remains poorly documented.

RRD without clinically evident complete PVD has been most commonly described in younger, myopic individuals and is typically associated with atrophic retinal holes or retinal dialysis rather than tractional flap tears. Noori et al. [3] reported RRD in patients without clinically evident complete PVD, operationally defined by the absence of a Weiss ring or a visible posterior hyaloid surface on biomicroscopy; this group predominantly comprised younger, phakic, and myopic patients with atrophic retinal holes or retinal dialysis. In that study, the presence of a horseshoe retinal tear was regarded as evidence of vitreoretinal traction. The present case differs from this more familiar phenotype because the patient was older, nonmyopic, and had a horseshoe retinal tear without clinically evident complete PVD. Posterior hyaloid attachment on posterior‐pole OCT does not establish the complete anatomical absence of vitreous separation because conventional scans cannot characterize the peripheral vitreoretinal interface or overall PVD configuration. Wide‐angle and widefield OCT studies have demonstrated partial vitreous separation outside standard macular scans, even when the vitreous appears attached within the posterior‐pole region [7–9].

The longitudinal observations directly document the clinical course of the detachment. At baseline, a localized macula‐on RRD associated with the horseshoe retinal tear was retrospectively identifiable on UWF imaging. Approximately 6 months later, the detachment had progressed to a bullous macula‐off RRD arising from the same tear. This sequence establishes progression over time but does not, by itself, identify the vitreoretinal forces responsible for tear formation or enlargement. The observed course is nevertheless compatible with clinically significant peripheral traction occurring before complete PVD becomes clinically evident.

A useful interpretive framework is that the vitreoretinal interface is better understood as a continuum rather than as a binary state of completely attached versus completely detached vitreous. Age‐related PVD evolves gradually through progressive vitreous liquefaction and vitreoretinal separation, with residual vitreoretinal adhesions persisting during its early or partial stages [10]. Recent OCT‐based staging studies have shown that PVD progresses through intermediate stages and that tractional vitreoretinal disorders are frequently associated with stages preceding complete separation [11]. Peng et al. [12] also reported an association between OCT‐defined Stage 3 PVD and horseshoe retinal tears in fellow eyes, supporting the possibility that clinically important tractional events may occur before complete posterior hyaloid separation. However, these associations do not establish that an incomplete or evolving PVD caused the horseshoe retinal tear in the affected eye of the present patient. The partial PVD observed in the fellow eye in the present case demonstrates interocular asymmetry in vitreous status but does not establish the precise stage or configuration of PVD in the affected eye.

Symptomatic horseshoe retinal tears are associated with persistent vitreoretinal traction and carry a substantial risk of progression to retinal detachment if left untreated [13, 14]. Within this clinical and anatomical framework, we hypothesize that age‐related vitreous liquefaction, an incomplete or evolving stage of PVD, and persistent focal vitreoretinal adhesion adjacent to the tear may have acted together to produce localized tractional stress. This proposed configuration was not directly demonstrated because the peripheral vitreoretinal interface adjacent to the horseshoe retinal tear was not imaged. It, therefore, represents a possible mechanistic interpretation rather than a confirmed explanation of tear formation in this patient.

The interval between the baseline localized macula‐on RRD and the subsequent bullous macula‐off RRD is also notable. At baseline, the subretinal fluid had extended posteriorly to within approximately 2.5 disc diameters of the fovea, yet the macula remained attached and central visual acuity was preserved. Progression to bullous macula‐off RRD was documented approximately 6 months later. Because imaging was available at only these two time points, it is not possible to determine whether the detachment enlarged gradually throughout the interval or remained relatively stable before more rapid progression. A recent review has emphasized that the interval between retinal tear formation and progression to RRD may provide an opportunity for timely detection and preventive treatment when a tear is recognized before detachment develops [1]. Although localized macula‐on RRD was already present at baseline in our patient, the preserved macular attachment and central visual acuity represented a clinically important opportunity for recognition and intervention before macular involvement. The observed interval, therefore, underscores the clinical importance of timely peripheral retinal evaluation but should not be considered direct evidence of intermittent or fluctuating traction.

Vitreous syneresis and intravitreal fluid dynamics provide a mechanistically plausible but unconfirmed basis for the proposed hypothesis. The coexistence of gel and liquefied phases creates structural and rheological heterogeneity within the vitreous cavity. Computational simulations of saccadic eye movements have demonstrated distinct flow behavior between vitreous gel and liquid phases, with vitreous velocity and wall shear stress increasing as saccadic displacement increases [15]. In a separate idealized model of partially liquefied vitreous, flow preferentially converged toward low‐resistance liquefied regions, resulting in localized amplification of flow velocity [16]. Together, these models demonstrate the physical plausibility of spatially heterogeneous intravitreal flow but do not establish the magnitude or distribution of traction transmitted to the retinal surface in this patient. In the presence of persistent focal vitreoretinal adhesion, such heterogeneous mechanical forces could theoretically contribute to localized tractional stress. Dynamic interactions within a partially liquefied vitreous should, therefore, be regarded as one possible explanatory framework rather than a causal conclusion.

This case highlights a clinically relevant diagnostic pitfall. At baseline, central visual acuity was 20/20 and posterior‐pole findings appeared reassuring, yet retrospective review of the UWF images demonstrated a temporal horseshoe retinal tear with localized macula‐on RRD. Preserved central visual acuity, unremarkable posterior‐pole findings, and absence of clinically evident complete PVD, therefore, should not reduce concern for peripheral vitreoretinal traction, associated retinal breaks, or RRD in symptomatic patients. Although UWF imaging improves visualization of the peripheral retina, peripheral retinal lesions may still be overlooked despite the wider field of view [17]. UWF imaging should, therefore, complement, rather than replace, meticulous dilated peripheral retinal evaluation, and the peripheral UWF image—particularly the temporal retina—should be intentionally reviewed when symptoms suggest vitreoretinal traction.

Several limitations should be acknowledged. First, this is a single case observation and, therefore, cannot establish generalizability or causality. Second, no targeted peripheral OCT assessment of the vitreoretinal interface adjacent to the horseshoe retinal tear was available. Although posterior‐pole OCT showed posterior hyaloid attachment at the macula and optic nerve head, localized peripheral vitreous separation, persistent focal adhesion, or an intermediate or evolving stage of PVD could not be excluded or anatomically characterized. Third, the available operative record documented intraoperative PVD induction but did not provide detailed information regarding the pre‐existing configuration of the peripheral vitreoretinal interface. Postoperative anatomical and visual outcomes were also unavailable because the patient underwent surgery at another institution. Finally, the proposed mechanism is inferential and was not directly demonstrated by the available imaging. The possible contributions of vitreous liquefaction, focal adhesion, and heterogeneous intravitreal fluid dynamics should, therefore, be regarded as a hypothesis‐generating framework rather than a causal conclusion.

In conclusion, this case provides longitudinal documentation of horseshoe retinal tear–associated RRD progressing from localized macula‐on to bullous macula‐off detachment over approximately 6 months in an older, nonmyopic eye without clinically evident complete PVD. Although the peripheral vitreoretinal interface was not directly imaged and the proposed mechanism remains inferential, the observed course is compatible with clinically significant focal traction occurring during an incomplete or evolving stage of PVD. Clinically, preserved central visual acuity, reassuring posterior‐pole findings, and absence of clinically evident complete PVD do not exclude peripheral horseshoe retinal tears or RRD in symptomatic patients. Meticulous dilated peripheral retinal examination and intentional review of peripheral UWF findings, therefore, remain essential for timely diagnosis.

## Author Contributions

**Donghyun Lee:** conceptualization, data curation, formal analysis, investigation, methodology, visualization, writing – original draft, writing – review and editing.

## Funding

No funding was received for this manuscript.

## Consent

Written informed consent was obtained from the patient for publication of this case report and accompanying images.

## Conflicts of Interest

The author declares no conflicts of interest.

## Supporting information


**Supporting Information** Additional supporting information can be found online in the Supporting Information section. Video S1: Optical coherence tomography demonstrating posterior hyaloid attachment at the macula and optic nerve head region. Sequential posterior‐pole OCT B‐scans demonstrate posterior hyaloid attachment at the macula and optic nerve head region in the right eye, with no clinically evident complete posterior vitreous detachment within the scanned area.

## Data Availability

The data that support the findings of this study are available on request from the corresponding author. The data are not publicly available due to privacy or ethical restrictions.

## References

[1] Chronopoulos A. , Schutz J. S. , and Finger R. P. , Prevention of Rhegmatogenous Retinal Detachment, Survey of Ophthalmology. (2025) 70, no. 6, 1061–1066, 10.1016/j.survophthal.2025.04.006. 40339663

[2] Govetto A. , Sebag J. , Lucchini S. , Ballabio C. , Matteucci M. , Ranno S. , Carini E. , Virgili G. , Bacherini D. , and Radice P. , Imaging Rhegmatogenous Retinal Lesions and Peripheral Vitreoretinal Interface With Widefield Optical Coherence Tomography, Retina. (2024) 44, no. 2, 269–279, 10.1097/IAE.0000000000003946, 37856780. 37856780

[3] Noori J. , Bilonick R. A. , and Eller A. W. , Scleral Buckle Surgery for Primary Retinal Detachment Without Posterior Vitreous Detachment, Retina. (2016) 36, no. 11, 2066–2071, 10.1097/IAE.0000000000001075. 27172097

[4] Gonzales C. R. , Gupta A. , Schwartz S. D. , and Kreiger A. E. , The Fellow Eye of Patients With Phakic Rhegmatogenous Retinal Detachment From Atrophic Holes of Lattice Degeneration Without Posterior Vitreous Detachment, British Journal of Ophthalmology. (2004) 88, no. 11, 1400–1402, 10.1136/bjo.2004.043240, 15489481. 15489481 PMC1772383

[5] Kurobe R. , Hirano Y. , Ogura S. , Yasukawa T. , and Ogura Y. , Ultra-Widefield Swept-Source Optical Coherence Tomography Findings of Peripheral Retinal Degenerations and Breaks, Clinical Ophthalmology. (2021) 15, 4739–4745, 10.2147/OPTH.S350080, 34983997. 34983997 PMC8699765

[6] Bacherini D. , Rizzo C. , Vicini G. , Luciani D. , Vannozzi L. , Virgili G. , Giansanti F. , and Nicolosi C. , Characterization of Peripheral Retinal Degenerations and Rhegmatogenous Lesions Using Ultra-Widefield Swept Source OCT Integrated With a Novel Scanning Laser Ophthalmoscope, Diagnostics. (2025) 15, no. 22, 10.3390/diagnostics15222930, 41300954. PMC12650825 41300954

[7] Abraham J. R. and Ehlers J. P. , Posterior Vitreous Detachment: Methods for Detection, Ophthalmology Retina. (2020) 4, no. 2, 119–121, 10.1016/j.oret.2019.12.014. 32033710

[8] Tsukahara M. , Mori K. , Gehlbach P. L. , and Mori K. , Posterior Vitreous Detachment as Observed by Wide-Angle OCT Imaging, Ophthalmology. (2018) 125, no. 9, 1372–1383, 10.1016/j.ophtha.2018.02.039, 29631900. 29631900

[9] Chiku Y. , Hirano T. , Takahashi Y. , Tuchiya A. , Nakamura M. , and Murata T. , Evaluating Posterior Vitreous Detachment by Widefield 23-mm Swept-Source Optical Coherence Tomography Imaging in Healthy Subjects, Scientific Reports. (2021) 11, no. 1, 19754, 10.1038/s41598-021-99372-z, 34611277. 34611277 PMC8492648

[10] Johnson M. W. , Posterior Vitreous Detachment: Evolution and Complications of Its Early Stages, American Journal of Ophthalmology. (2010) 149, no. 3, 371–82.e1, 10.1016/j.ajo.2009.11.022, 20172065. 20172065

[11] Brown D. M. , Laswell S. M. , Rahman E. Z. , Fan K. C. , Shah A. , Patel S. B. , and Wykoff C. C. , Clinically Relevant Posterior Vitreous Detachment Staging Using Circumpapillary and Macular Volume Optical Coherence Tomography, Retina. (2024) 44, no. 8, 1441–1448, 10.1097/IAE.0000000000004115, 39047131. 39047131 PMC11280448

[12] Peng E. T. , Parvus M. N. , Yu H. J. , Laswell S. M. , Pearce W. A. , Fan K. C. , Major J. C. , Brown D. M. , Wykoff C. C. , and Patel S. B. , Risk Factors for the Development of Fellow Eye Horseshoe Retinal Tears Following Horseshoe Retinal Tear in the Presenting Eye, Ophthalmic Surgery, Lasers and Imaging Retina. (2023) 54, no. 6, 338–345, 10.3928/23258160-20230523-01, 37352399. 37352399

[13] Colyear B. H. and Pischel D. K. , Preventive Treatment of Retinal Detachment by Means of Light Coagulation, Transactions of the Pacific Coast Oto-Ophthalmological Society Annual Meeting. (1960) 41, 193–217, 13694878. 13694878

[14] Shea M. , Davis M. D. , and Kamel I. , Retinal Breaks Without Detachment, Treated and Untreated, Modern Problems in Ophthalmology. (1974) 12, 97–102, 4422249. 4422249

[15] Silva A. F. , Pimenta F. , Alves M. A. , and Oliveira M. S. N. , Flow Dynamics of Vitreous Humour During Saccadic Eye Movements, Journal of the Mechanical Behavior of Biomedical Materials. (2020) 110, 103860, 10.1016/j.jmbbm.2020.103860, 32755799. 32755799

[16] Khoobyar A. , Penkova A. , Humayun M. S. , Irimia A. , and Sadhal S. S. , Flow Characterization in a Partially Liquefied Vitreous Humor, Transport in Porous Media. (2024) 151, no. 3, 533–558, 10.1007/s11242-023-02052-x, 39391233. 39391233 PMC11466277

[17] Lee D. H. , Kim S. S. , Kim M. , and Koh H. J. , Identifiable Peripheral Retinal Lesions Using Ultra-Widefield Scanning Laser Ophthalmoscope and Its Usefulness in Myopic Patients, Journal of the Korean Ophthalmological Society. (2014) 55, no. 12, 1814–1820, 10.3341/jkos.2014.55.12.1814.

